# An exploration of service use pattern changes and cost analysis following implementation of community perinatal mental health teams in pregnant women with a history of specialist mental healthcare in England: a national population-based cohort study

**DOI:** 10.1186/s12913-024-10553-8

**Published:** 2024-03-20

**Authors:** Emma Tassie, Julia Langham, Ipek Gurol-Urganci, Jan van der Meulen, Louise M Howard, Dharmintra Pasupathy, Helen Sharp, Antoinette Davey, Heather O’Mahen, Margaret Heslin, Sarah Byford

**Affiliations:** 1 King’s Health Economics, Institute of Psychiatry, Psychology & Neuroscience, King’s College London, London, UK; 2 Department of Health Services Research & Policy, London School of Hygiene and Tropical Medicine, London, UK; 3 Section of Women’s Mental Health, Institute of Psychiatry, Psychology & Neuroscience, King’s College London, London, UK; 4 Reproduction and Perinatal Centre, Faculty of Medicine and Health, University of Sydney, Sydney, NSW Australia; 5 Department of Primary Care and Mental Health, University of Liverpool, Liverpool, UK; 6 Faculty of Life and Environmental Sciences, Department of Psychology, University of Exeter, Perry Road, Prince of Wales Road, Exeter, UK

**Keywords:** Costs, Health service use, Mental health, Perinatal

## Abstract

**Background:**

The National Health Service in England pledged >£365 million to improve access to mental healthcare services via Community Perinatal Mental Health Teams (CPMHTs) and reduce the rate of perinatal relapse in women with severe mental illness. This study aimed to explore changes in service use patterns following the implementation of CPMHTs in pregnant women with a history of specialist mental healthcare in England, and conduct a cost-analysis on these changes.

**Methods:**

This study used a longitudinal cohort design based on existing routine administrative data. The study population was all women residing in England with an onset of pregnancy on or after 1st April 2016 and who gave birth on or before 31st March 2018 with pre-existing mental illness (*N* = 70,323). Resource use and costs were compared before and after the implementation of CPMHTs. The economic perspective was limited to secondary mental health services, and the time horizon was the perinatal period (from the start of pregnancy to 1-year post-birth, ~ 21 months).

**Results:**

The percentage of women using community mental healthcare services over the perinatal period was higher for areas with CPMHTs (30.96%, n=9,653) compared to areas without CPMHTs (24.72%, n=9,615). The overall percentage of women using acute care services (inpatient and crisis resolution teams) over the perinatal period was lower for areas with CPMHTs (4.94%, n=1,540 vs. 5.58%, n=2,171), comprising reduced crisis resolution team contacts (4.41%, n=1,375 vs. 5.23%, n=2,035) but increased psychiatric admissions (1.43%, n=445 vs. 1.13%, n=441). Total mental healthcare costs over the perinatal period were significantly higher for areas with CPMHTs (fully adjusted incremental cost £111, 95% CI £29 to £192, *p*-value 0.008).

**Conclusions:**

Following implementation of CPMHTs, the percentage of women using acute care decreased while the percentage of women using community care increased. However, the greater use of inpatient admissions alongside greater use of community care resulted in a significantly higher mean cost of secondary mental health service use for women in the CPMHT group compared with no CPMHT. Increased costs must be considered with caution as no data was available on relevant outcomes such as quality of life or satisfaction with services.

**Supplementary Information:**

The online version contains supplementary material available at 10.1186/s12913-024-10553-8.

## Background

Perinatal mental illness (mental illness occurring during pregnancy or the year after childbirth) is estimated to affect 10–20% of women, is associated with increased morbidity and is a leading cause of maternal death during the perinatal period [1–5]. Evidence suggests that women with pre-existing mental illness before the onset of pregnancy have an increased risk of adverse maternal and neonatal outcomes such as stillbirth, preterm delivery, and low birth weight babies [5–7]. Perinatal mental illness can have long-lasting negative impacts on the woman, the baby, the immediate and wider family and incur substantial societal costs [4, 8]. The estimated cost to society of perinatal depression, anxiety and psychosis is £8.1 billion for each annual cohort of births in the UK, with 72% of this cost attributable to the additional long-term healthcare needs of children born to women with perinatal mental illness [4, 9].

In 2014, the National Institute for Health and Care Excellence (NICE) published recommendations that women who have, are suspected of having, or have a family history of serious perinatal mental illness should be referred to secondary mental health services, preferably those specialising in perinatal mental health, such as community perinatal mental teams (CPMHTs) [10]. CPMHTs are multi-disciplinary teams which, in the UK, are defined as consisting of a minimum of a psychiatrist, a psychologist and a specialist nurse specialising in perinatal mental health.

In 2016, the Mental Health Taskforce published several recommendations to improve perinatal mental health services in England, including that at least 30,000 more women each year should have access to evidence-based specialist perinatal mental health services [11]. In response, the National Health Service (NHS) in England (NHS England) pledged £365 million over five years (2016–2021) (and more in subsequent years) to provide timely and equitable access to CPMHTs to improve the lives of women and their families. This included improving access to perinatal mental healthcare and reducing the risk of postpartum relapse in women with severe mental illness (which would therefore potentially reduce the use of acute care services such as inpatient treatment and crisis resolution teams). Furthermore, following a psychiatric admission during the perinatal period, women would have follow-up by a specialist community team [12].

This study aimed to explore changes in patterns of service use and the cost of that service use by pregnant women with a history of specialist mental healthcare in England following the implementation of CPMHTs. We hypothesised that areas with CPMHTs would be associated with lower rates of acute care (defined as psychiatric admissions or crisis resolution team (CRT) contacts during the perinatal period), shorter duration of admissions, lower rates of Mental Health Act detention, and lower cost than areas without CPMHTs.

## Methods

### Study design

This study used a longitudinal cohort design based on existing data (described below) to explore changes in service use patterns and the cost of that service use before and after the implementation of CPMHTs. The economic perspective was limited to secondary mental health services, and the time horizon was the perinatal period (from the estimated start of pregnancy to 1-year post birth, approximately 21 months). Given the focus of CPMHTs on secondary mental healthcare, the perspective does not include primary care, attendances at accident and emergency or general hospital admissions.

### Data sources and linkage

Data were taken from the Mental Health Services Dataset (MHSDS), Hospital Episode Statistics (HES), and Personal Demographic Service (PDS) Birth Notification Data [13–15]. The MHSDS (formally known as the Mental Health Minimum Dataset (MHMDS) and the Mental Health and Learning Disabilities Dataset (MHLDDS)), collects individual level data on people who are in contact with mental health services. It includes all activity relating to patients who receive assessments and treatment from Mental Health Services in England. As the MHSDS is an administrative dataset, several challenges with retrieving the length of hospital admission and cluster assignment (process of classifying patients into care clusters based on their level of need and complexity) data were encountered, such as data ambiguities and missingness. Several data-cleaning assumptions were required (see supplementary material 1). The HES dataset provides information on healthcare activity, including patient demographics, hospital admissions, diagnoses (coded using ICD-10) and procedures (coded using OPCS-4) [16]. Each birth event in the HES includes a non-mandatory ‘maternity tail’, which records more detailed information about the pregnancy and childbirth, such as gender, gestational age, stillbirth and birth weight. The PDS is used by the NHS to manage patient demographic data. The PDS Birth Notification Data is a subset of the PDS which records information on maternity outcomes of all newborn babies. The PDS Birth Notification Data also includes the mother’s NHS number, which enables linkage to other national electronic health datasets. Where data was missing from HES, the PDS Birth Notification data was used.

The MHSDS was combined at the patient level to HES and PDS Birth Notification between 1st April 2016 and 31st March 2019 to generate the study cohort and provide mental health service use and outcome data for the perinatal period for all women in the cohort. The linkage between the datasets was undertaken by NHS Digital using their standard deterministic linkage protocol based on the mother’s NHS number.

### Study population

The study population was all women residing and giving birth in England with an onset of pregnancy on or after the 1st April 2016 and who gave birth on or before 31st March 2018 with pre-existing mental illness (defined as those with a previous secondary mental health care contact). Data from HES and the PDS Birth Notification Dataset were used to identify women who gave birth during the study period. Women were considered to have a pre-existing mental illness if they had used secondary mental healthcare services (captured in the MHSDS, MHMDS and MHLDDS) in the ten years before the onset of pregnancy. For women with two or more births during the study period, one birth (randomly selected) was included. The following exclusions were applied to the cohort: age less than 18 years, multiple births, and gestation period less than 24 weeks.

### Intervention

The intervention was access to a CPMHT during the perinatal period and was compared to no access to a CPMHT. Women were considered to have had access to a CPMHT if the date of CPMHT implementation in their region of residence was before the onset of pregnancy. Data on CPMHT status was assessed at the clinical commissioning group (CCG) level and was based on the availability of at least a dedicated psychiatrist, psychologist and a specialist nurse in place, as recommended by commissioning guidance [17, 18]. CPMHTs are a service for women with mental health problems, who are planning a pregnancy, pregnant or who have a baby up to one year old. They aim to help women stay mentally well during this time, and support women who become unwell [19].

CCGs were established in 2013 as clinically led NHS bodies responsible for the planning and commissioning of health care services for their local area until July 2022. There were 211 CCGs initially established in 2013, but through mergers, this number reduced to 135 by 2020. Prior to 2016, few CPMHTs existed in England. In April 2016 (first pregnancies in this cohort), CPMHTs were in operation in 81 out of 207 CCGs (35%), increasing to 135 out of 207 (65%) in October 2017 (last pregnancy month in this cohort) [17].

### Service use measurement

The MHSDS provided patient-level data on contacts with secondary mental health services, including: (i) acute care, consisting of psychiatric inpatient stays and crisis resolution team (CRT) contacts (specialist community mental health teams that provide timely assessment and comprehensive intensive treatment in the home of a person experiencing a mental health crisis); and (ii) other community mental health care (referred to as ‘community care’), consisting of any other care contact with secondary mental healthcare services (including contacts with community mental health teams, early intervention teams for psychosis, perinatal mental illness services, general psychiatric services, etc.).

Psychiatric inpatient service use was measured as the number of admitted days using Table 501 Hospital Provider Spell from the MHSDS dataset. The length of a hospital stay was calculated by cumulating the number of days between the admission and discharge dates for each admission occurring during the perinatal period. CRT and community mental health contacts were derived from Table 201 Care Contact, which provided the date of all care contacts, and linked to Table 6 Mental Health Care Coordinator, to provide the type of contact (i.e., CRT or other community care). Detentions under the Mental Health Act were retrieved from Table MHS401 (Mental Health Act Legal Status Classification Period) of the MHSDS. A binary outcome was coded for women who had a formal detention (1) and those who had an informal detention or were not detained (0) under the Mental Health Act.

### Service use valuation

#### Psychiatric admitted days

Inpatient admissions were costed using a mental health cluster framework which firstly involved combining the number of admitted days with mental health care cluster assignment. Mental health care clusters are used to categorise patients into one of 21 mutually exclusive clusters that determine the package of care a patient receives and the payment providers receive [20, 21]. The clusters are conceptually made up of three super clusters: non-psychotic (clusters 1 to 8), psychotic (clusters 10 to 17) and organic (clusters 18 to 21) [20, 21]. Cluster 9 is intentionally left blank and is not used; cluster 0 is a variance cluster that is used for patients who cannot be categorised into one of the other clusters but requires mental health support, and cluster 99 is used when the patient has not been assessed or assigned a mental health cluster.

The overlapping dates between hospital admissions and cluster-episode information were used to derive the length of hospital admission in a mental health cluster, $$ {admitteddays}_{c}$$. Unit costs for $$ {admitteddays}_{c}$$ in a care cluster were retrieved from the NHS Reference Costs [22]. The NHS Reference Cost data provide national average cost information on admitted days in a cluster that varies by the 21 mental health clusters in its classification system. All costs were reported in pounds sterling at 2018/2019 prices. The length of hospital admission $$ {admitteddays}_{c}$$ was multiplied by the appropriate mental health cluster reference unit cost per occupied bed day $$ {rc}_{adm,c}$$ (see supplementary material 1). Where the mental health care cluster was missing, mental health care cluster 99 was applied. The cluster cost, $$ {C}_{c}$$, is, denoted as follows:

Eq. 1 $$ {C}_{c}= {admitteddays}_{c}*{rc}_{adm,c}$$

Although clusters are mutually exclusive, women could be assigned to more than one cluster at different times during the perinatal period. The total inpatient cost was the sum of all cluster costs, $$ {C}_{c}$$, occurring during the perinatal period.

#### CRT and community mental healthcare

Unit costs were applied to the number of CRT and community mental healthcare contacts occurring during the perinatal period for each woman (supplementary material 1). The unit cost applied for a CRT contact was taken from PSSRU (2016), CRT for adults with mental health problems, inflated to 2018/19 prices [23]. We assumed one hour with one community mental health team member for community contacts (inflated to 2018/19 prices) [24].

Mental Health Act detentions were not costed per se, since these detentions are encompassed in admitted days and other secondary mental health services. Discounting was not applied as costs and outcomes were evaluated for each woman within the perinatal period (~ 21 months) and it was not possible to clearly separate contacts into the first 12 months versus the last 9 months.

### Analyses

The datasets were combined, managed and analysed using STATA 17 [25]. Sociodemographic and clinical characteristics are described using mean/range and number/percentage as appropriate. Maternal age was reported as the mean age and a categorical variable with four categories (under 25, 25 to 34, 35 to 39 and 40 years and above). The Office for National Statistics (ONS) categorisation of ethnicity from the 2001 census was used to code maternal ethnicity using the following categories: White, South Asian, Black, Mixed and other stated. Socioeconomic deprivation was derived from the quintiles of the Index of Multiple Deprivation 2019 (IMD) national ranking. Parity was reported as nulliparous (no previous live births), multiparous (had previous live birth) with a caesarean section and multiparous without a caesarean section. Parity was obtained from the HES ‘maternity tail’. Data on pregnancy risk factors were derived from diagnosis codes in the HES maternal records; if there was no record, women were assumed not to have the pregnancy risk factor.

Generalised least squares (GLS) regression models were used to estimate the mean difference in mental health service use costs associated with regions with and without CPMHTs. GLS regressions were used to estimate the cost difference for acute care costs (inpatient stays and CRT contacts), community care costs and total costs. A GLS is a panel-data linear model that allows for autocorrelation within panels and cross-sectional correlation and heteroskedasticity across panels [26]. Cost models were estimated with random effects at the CCG level. To account for the skewed nature of healthcare costs, non-parametric bootstrapping with 5,000 estimates was used to generate the standard errors and 95% confidence intervals around the cost difference [27].

Three model specifications were conducted for cost data. Model 1 included a binary exposure variable (whether or not CPMHT was implemented) only. Model 2 included the binary exposure variable and a time variable defined as the month of onset of pregnancy in a linear form. This time variable controls for trends over time. Model 3 (the ‘fully adjusted’ model) included the binary exposure and time variables plus adjustments for sociodemographic characteristics (maternal age, ethnicity and socioeconomic deprivation), highest level of pre-pregnancy care contact (inpatient, CRT or community care), the timing of the most recent pre-pregnancy care contact (< 1 year, 1–5 years and > 5 years) and maternal risk factors (parity, pre-existing hypertension, pre-existing diabetes mellitus, pre-eclampsia and gestational diabetes).

We conducted two sensitivity analyses. Firstly, we used with multiple imputation by chained equations to assess whether missing data impacted the results. Significant predictors of missingness at the 10% level and all adjustment variables in Model 3 were included in the multiple imputation model. We used the ‘mi impute chained’ command in Stata version 17, with 10 imputed datasets estimated for each model. Secondly, we assessed the robustness of results to the exclusion of women who had an onset of pregnancy within the first six months after CPMHT was implemented. This analysis was undertaken to allow time to embed the CPMHT services in the area. GLS regression models were repeated to estimate the mean cost difference in mental health service use costs for acute care costs (inpatient stays and CRT contacts), community care costs and total costs.

Logistic regression was used to estimate odds ratios for formal detentions under the Mental Health Act, using the same three model specifications as for costs and with robust standard errors to account for any clustering outcomes within CCG [26].

## Results

### Cohort

Data from all women who gave birth in England between 1st December 2016 and 31st March 2018 were first extracted from HES (*n* = 807,798). The following exclusions were then applied: not resident in England (*n* = 1,665), age < 18 (*n* = 5,646), multiple births (*n* = 14,323) and gestational length < 24 weeks (*n* = 1,312). This resulted in 785,131 eligible maternity episodes. A further 5,105 births were excluded by randomly choosing one birth for women who had more than one birth during the study period. A further 709,703 women who did not have a pre-pregnancy mental health contact in the preceding 10 years were excluded, resulting in an eligible cohort of 70,323 women. We had complete economic data for 70,082 women (99.7%).

There was a good balance between those with and without CPMHTs implemented on most sociodemographic and pregnancy risk factors (Table 1). The majority of women were under 35 years old (~ 80%) and from a White ethnic background (78%). The highest proportion of women resided in areas of the most deprived socioeconomic quintile (5) with the proportion of women in each quintile decreasing as the quintile decreases. Most women had given birth before without a caesarean section (50%), followed by women who had not given birth (32%) and women who had given birth with a caesarean section (12%). The majority of women (76%) had only had community mental healthcare in the past, with almost 20% having crisis resolution team contact, and 5% having used inpatient services. Almost 50% of women had had contact with mental health services 1–5 years ago, with approximately a quarter having contact both less than a year ago, and a quarter having contact more than 5 years ago. Differences in sociodemographic and clinical characteristics between the full economic cohort and those without full economic data are described in the supplementary material.

**Table 1:** Sociodemographic characteristics, pregnancy risk factors and pre-pregnancy contacts

Characteristic	Full sample (*N* = 70,323)	No CPMHT (*n* = 38,901)	CPMHT (*n* = 31,181)
Maternal age categories n (%)			
18 to 24	17,463 (24.83)	9,936 (25.54)	7,468 (23.95)
25 to 34	40,031 (56.92)	22,142 (56.92)	17,770 (56.99)
35 to 39	10,216 (14.53)	5,464 (14.05)	4,703 (15.08)
40 and over	2,607 (3.71)	1,355 (3.48)	1,238 (3.97)
Missing	6 (0.01)	4 (0.01)	2 (0.01)
Obstetric history n (%)			
Nulliparous	22,665 (32.23)	12,691 (32.62)	9,877 (31.68)
Multiparous, no previous CS	35,423 (50.37)	19,493 (50.11)	15,822 (50.74)
Multiparous, previous CS	8,646 (12.29)	4,688 (12.05)	3,939 (12.63)
Missing	3,589 (5.10)	2,029 (5.22)	1,543 (4.95)
Ethnicity n (%)			
White	54,965 (78.16)	31,096 (79.94)	23,712 (76.05)
South Asian	3,244 (4.61)	1,609 (4.14)	1,615 (5.18)
Black	1,779 (2.53)	683 (1.76)	1,078 (3.46)
Mixed	1,332 (1.89)	659 (1.69)	669 (2.15)
Other stated	1,160 (1.65)	521 (1.34)	630 (2.02)
Missing	7,843 (11.15)	4,333 (11.14)	3,477 (11.15)
Socioeconomic deprivation n (%)			
Quintile 1 (least deprived)	7,373 (10.48)	4,257 (10.94)	3,096 (9.93)
Quintile 2	9,765 (13.89)	5,514 (14.17)	4,224 (13.55)
Quintile 3	12,560 (17.86)	6,802 (17.49)	5,716 (18.33)
Quintile 4	16,522 (23.49)	8,464 (21.76)	8,009 (25.69)
Quintile 5 (most deprived)	24,100 (34.27)	13,862 (35.63)	10,135 (32.50)
Missing	3 (0.00)	2 (0.01)	1 (0.00)
Pregnancy risk factors n (%)			
Pre-existing diabetes	1,021 (1.45)	498 (1.28)	517 (1.66)
Missing	3,589 (5.10)	2,029 (5.22)	1,543 (4.95)
Pre-existing hypertensive conditions	488 (0.69)	250 (0.64)	237 (0.76)
Missing	3,589 (5.10)	2,029 (5.22)	1,543 (4.95)
Gestational diabetes	4,294 (6.11)	2,252 (5.79)	2,021 (6.48)
Missing	3,589 (5.10)	2,029 (5.22)	1,543 (4.95)
Pre-eclampsia	1,442 (2.05)	776 (1.99)	658 (2.11)
Missing	3,589 (5.10)	2,029 (5.22)	1,543 (4.95)
Highest level of pre-pregnancy contact n (%)			
Psychiatric inpatient	3,272 (4.67)	1,845 (4.74)	1,427 (4.58)
Crisis resolution team	13,776 (19.66)	8,207 (21.10)	5,569 (17.86)
Community healthcare	53,034 (75.67)	28,849 (75.16)	24,185 (77.56)
Timing of most recent pre-pregnancy contact n (%)			
> 5 years	18,292 (26.10)	10,213 (26.25)	8,079 (25.91)
1–5 years	34,612 (49.39)	19,740 (50.74)	14,872 (47.70)
< 1 years	17,178 (24.51)	8,948 (23.00)	8,230 (26.39)

### Service use and costs

Table 2 shows the service use and cost summary statistics. During the follow-up period, 5.30% of women in the cohort (*n* = 3,711) had contact with acute care services (psychiatric inpatient stays and crisis resolution team contacts) over the perinatal period. This was lower for women with access to CPMHTs (4.94%; *n* = 1,540) versus no CPMHT access (5.58%; *n* = 2,171). When acute care was broken down into its two components, 1.26% (*n* = 886) of women in the cohort had at least one psychiatric inpatient hospital stay over the perinatal period and 4.87% (*n* = 3,410) had at least one CRT contact. Inpatient use was higher for women who had access to CPMHTs (1.43%; *n* = 445 ) versus those without access (1.13%; *n* = 441). CRT use was lower for women who had access to CPMHTs (4.41%; *n* = 1,375) versus those without (5.23%; *n* = 2,035). In the full sample, 27.49% (*n* = 19,268) had at least one contact with other community care services. This was higher for women who had access to CPMHTs (30.96%; *n* = 9,653) versus those without (24.72%; *n* = 9,615).

In terms of mean use of these services over the perinatal period, women who had access to CPMHTs compared to no CPMHT had higher numbers of acute care contacts in total (mean 1.11 versus 0.87 contacts), longer lengths of inpatient stays (mean 0.85 versus 0.55 days), lower numbers of CRT contacts (mean 0.27 versus 0.32) and higher numbers of community care contacts (mean 4.31 versus 3.26 contacts).

In terms of mean costs over the perinatal period, women who had access to CPMHTs compared with no CPMHT had higher acute care costs (mean £384 versus £282), higher inpatient costs (mean £332 versus £218), lower CRT costs (mean £54 versus £64) and higher community healthcare costs (mean £174 vs. £132). Overall, total costs over the perinatal period were higher for areas with CPMHTs in place compared to those without (mean £561 versus £414).

**Table 2:** Service use and cost summary statistics

Service	Full sample (*n* = 70,082)	No CPMHT (*n* = 38,901)	CPMHT (*n* = 31,181)
**Number using each service n (%)**			
Total acute care	3,711 (5.30)	2,171 (5.58)	1,540 (4.94)
Psychiatric admission	886 (1.26)	441 (1.13)	445 (1.43)
Crisis resolution team	3,410 (4.87)	2,035 (5.23)	1,375 (4.41)
Community care	19,268 (27.49)	9,615 (24.72)	9,653 (30.96)
**Number of days/contacts mean (SD); range**			
Total acute care	0.98 (10.40); 0 to 461	0.87 (9.39); 0 to 461	1.11 (11.53); 0 to 446
Psychiatric admitted days	0.68 (9.50); 0 to 455	0.55 (8.49); 0 to 455	0.85 (10.62); 0 to 446
Crisis resolution team contacts	0.29 (2.11); 0 to 107	0.32 (2.16); 0 to 100	0.27 (2.04); 0 to 107
Community care contacts	3.73 (11.40); 0 to 241	3.26 (10.75); 0 to 202	4.31 (12.14); 0 to 241
**Costs mean (SD); range**			
Total acute care	£328 (3,994); 0 to 210,024	£282 (3,615); 0 to 210,024	£387 (4,421); 0 to 177,702
Psychiatric admission	£269 (3,834); 0 to 208,813	£218 (3,459); 0 to 208,813	£332 (4,245); 0 to 176,254
Crisis resolution team	£60 (426); 0 to 21,600	£64 (437); 0 to 20,187	£54 (412); 0 to 21,600
Community care	£150 (460); 0 to 9,727	£132 (434); 0 to 8,153	£174 (490); 0 to 9,727
Total	£479 (4,196); 0 to 213,455	£414 (3,807); 0 to 213,455	£561 (4,634); 0 to 182,506

Table 3 shows the results of the generalised least squares (GLS) regression models used to estimate the mean difference in secondary mental health resource use costs for regions with CPMHTs in place, compared to regions without. Acute care costs were significantly higher for areas with CMPHTs implemented compared to those without in the final adjusted model (fully adjusted mean difference in cost £80, 95% CI £4 to £157, *p*-value 0.0140). When acute care is broken down into its two components, costs were significantly higher for CPMHT compared to no CPMHT for psychiatric inpatient stays (fully adjusted mean difference £90, 95% CI £18 to £163, *p*-value 0.015), but not for CRT contacts (fully adjusted mean difference -£11, 95% CI -£22 to £0, *p*-value 0.058). Community mental healthcare costs were also significantly higher for women in areas with CPMHTs implemented (fully adjusted mean difference £22, 95% CI £10 to £34, *p*-value < 0.001). Total costs were significantly higher for women in areas with CPMHTs implemented (fully adjusted incremental cost £111, 95% CI £29 to £192, *p*-value 0.008).

**Table 3:** Generalised least squares (GLS) regression models for differences in cost for CPMHT versus no CPMHT

**Service**	Model 1			Model 2			Model 3		
	**Cost diff £ (SE)**	**95% CI**	*P*-value	**Cost diff £ (SE)**	**95% CI**	*P*-value	**Cost diff £ (SE)**	**95% CI**	*P*-value
Total acute care	109 (41)	28 to 190	0.008	103 (43)	20 to 188	0.016	80 (39)	4 to 157	0.040
Psychiatric admissions	119 (40)	40 to 197	0.003	114 (40)	35 to 193	0.005	90 (37)	18 to 163	0.015
Crisis resolution team	-8 (6)	-20 to 4	0.210	-9 (6)	-21 to 2	0.123	-11 (6)	-22 to 0	0.058
Community care	25 (7)	12 to 39	0.000	21 (7)	7 to 35	0.003	22 (6)	10 to 34	0.000
Total	115 (46)	55 to 234	0.002	137 (48)	43 to 231	0.004	111 (42)	29 to 192	0.008

Model 1: binary exposure variable; Model 2: binary exposure and time trend variable; Model 3: fully adjusted model

### Sensitivity analysis

The multiple imputation analyses for missing data show negligible changes to cost differences that do not change the conclusions of the full case analysis, although the difference in acute care costs became non-significant (fully adjusted mean difference £56, 95% CI -£8 to £120, *p*-value 0.087) (Table 4). Table 5 shows the results of the sensitivity analysis excluding women who had an onset of pregnancy within the first six months of CPMHT implementation. The results are largely consistent with the full case analysis, except for the significance of total acute care costs which became non-significant (fully adjusted mean difference £73, 95% CI -£2 to £149, p-value 0.059).

**Table 4:** Sensitivity analysis: generalised least squares (GLS) regression models for differences in cost for CPMHT versus no CPMHT with multiple imputation for missing data

	Model 1			Model 2			Model 3		
	**Cost diff £ (SE)**	**95% CI**	*P*-value	**Cost diff £ (SE)**	**95% CI**	*P*-value	**Cost diff £ (SE)**	**95% CI**	*P*-value
Total acute care	100 (41)	17 to 175	0.018	100 (41)	16 to 176	0.019	56 (33)	-8 to 120	0.087
Psychiatric admissions	107 (39)	32 to 183	0.005	108 (39)	31 to 184	0.006	69 (31)	8 to 130	0.027
Crisis resolution team	-9 (6)	-21 to 3	0.142	-10 (6)	-22 to 2	0.117	-11 (6)	-22 to 0.08	0.052
Community care	26 (7)	13 to 40	0.000	24 (7)	10 to 37	0.000	23 (5)	13 to 34	0.000
Total	132 (44)	45 to 220	0.003	131 (45)	42 to 219	0.004	87 (35)	19 to 155	0.012

Model 1: binary exposure variable; Model 2: binary exposure and time trend variable; Model 3: fully adjusted model

**Table 5:** Sensitivity analysis: generalised least squares (GLS) regression models for differences in cost for CPMHT versus no CPMHT excluding women with an onset of pregnancy within the first six months after CPMHT was implemented

**Service**	Model 1			Model 2			Model 3		
	**Cost diff £ (SE)**	**95% CI**	*P*-value	**Cost diff £ (SE)**	**95% CI**	*P*-value	**Cost diff £ (SE)**	**95% CI**	*P*-value
Total acute care	101 (43)	15 to 186	0.021	100 (44)	11 to 183	0.028	73 (39)	-2 to 149	0.059
Psychiatric admissions	111 (41)	30 to 192	0.007	108 (42)	25 to 190	0.010	83 (37)	11 to 156	0.025
Crisis resolution team	-9 (7)	-22 to 5	0.206	-10 (7)	-23 to 4	0.162	-11 (6)	-23 to 0.30	0.056
Community care	30 (7)	16 to 45	0.000	26 (8)	11 to 41	0.001	26 (6)	13 to 38	0.000
Total	137 (47)	44 to 230	0.004	131 (49)	36 to 226	0.007	103 (41)	23 to 184	0.012

Model 1: binary exposure variable; Model 2: binary exposure and time trend variable; Model 3: fully adjusted model

### Mental health act detentions

The odds of a formal detention under the Mental Health Act for women with access to CPMHTs versus women with no access to CPMHTs was not statistically different (fully adjusted OR 0.926, 95% CI 0.760 to 1.130, *p*-value 0.444; unadjusted OR 1.086, 95% CI 0.883 to 1.335, *p*-value 0.437).

## Discussion

### Summary of results

In this study, we explored service use pattern and cost changes following implementation of CPMHTs for pregnant women with a history of secondary mental healthcare use in England. The results suggest that a lower proportion of women with access to CPMHTs compared to those with no CPMHT had contact with acute care services (admissions and CRTs), a higher proportion had contact with community care services, and, overall, a higher proportion had contact with any secondary mental health services. These results suggest that the implementation of CPMHTs has supported increased access to secondary mental healthcare services, particularly community mental healthcare.

Whilst the percentage of women accessing acute care was lower overall in the CPMHT group, supporting our hypothesis that areas with CPMHTs would be associated with lower rates of acute care in the perinatal period, this was driven by lower use of CRTs. In terms of psychiatric inpatient services, a higher proportion of women in the CPMHT group were admitted and, if admitted, they spent longer in those services, which was not in the hypothesised direction. This may reflect better identification and admission of more severely unwell women, requiring more intensive support over longer time periods, with better support being provided in the community for women less severely unwell. It may also reflect greater access in CPMHT regions to mother and baby units (MBUs) where admissions are typically longer than for traditional psychiatric wards [28]. Length of admission is influenced by many factors, including severity of illness as well as availability of support in the community. However, a key factor in perinatal mental health populations is whether women are together with or separated from their baby. In traditional psychiatric wards, there is likely to be a greater sense of urgency to discharge women so they can be returned to their baby, which is not felt in MBUs where women are able to keep their babies with them.

This greater use of inpatient admissions alongside greater use of community care resulted in a significantly higher mean cost of secondary mental health service use over the perinatal period for women in the CPMHT group compared with no CPMHT, which was not in the hypothesised direction. However, from a service provision perspective, the increased cost could be reflective of patients receiving targeted specialised mental healthcare during the perinatal period. Furthermore, the results did not support the hypothesis that access to CPMHTs would lead to reductions in formal detentions under the Mental Health Act, with no differences between the groups.

### Strengths and limitations

This study has several strengths: using linked national datasets provided a large number of patient records of care provided by the NHS. The large cohort of women with pre-existing mental illness provided greater precision in the cost estimates. The NHS provides the majority of secondary mental healthcare in England meaning a high coverage for this dataset. Further, we used contact with secondary mental health services to identify women with severe mental illness, rather than relying on recorded mental health diagnoses which can be recorded inconsistently and incompletely in administrative datasets [29]. Additionally, we assigned women to CPMHTs or not, based on the availability of a CPMHT in their region in 2016-8 rather than based on whether women actually received care from CPMHTs. This is a strength because it reduces bias that may come when decisions are being made about providing care through CPMHTs and other factors which may influence that, because there is no apparent direct link between a women’s characteristics and whether CPMHTs are available in her area of care.

This study also has several limitations. Routine healthcare datasets come with limitations concerning variable availability and data quality. Within this dataset, we were missing four-months of ‘look-back’ data (during the change-over to MHSDS) for previous mental health contacts. If a woman had her only contact with mental health services during this time, she will not be included in our cohort. However, it is unlikely many women will have been missed because of this given the relatively small gap in the context of the 10-year ‘look-back’ period and the fact that we would expect most women to have multiple contacts with mental health services. Other issues related to data availability and quality including a shorter ‘look-back’ period for younger women as we did not have access to child/adolescent mental health care contacts therefore we only had full 10-year look-back data for women aged 28 or over. In addition, as our study explored the impact of CPMHTs for women with a pre-existing mental illness, women who had a first contact with mental health services during pregnancy or one year following pregnancy were not included in the cohort. Further, as discussed in the methods, as an administrative dataset, the MHSDS data required data-cleaning assumptions to be able to use it, and in terms of CPMHT availability or not, we used a single date to determine this, without being able to account for the time it may take to fully implement a CPMHT and embed it into the network of services and referral agencies within the area. It is possible that the impact of CPMHTs on the pattern of service use and costs may develop further in the future.

Finally, while this study’s findings suggest that total mental healthcare costs increased in areas with CPMHTs, we were unable to measure any potential reduction in other costs (e.g. general hospital admissions, accident and emergency attendances or primary care use), and we were unable to estimate changes in important patient outcomes such as quality of life, or service satisfaction. Perinatal mental illness affects more than just the woman and can have long-lasting consequences for the baby, immediate and wider family and costs to society. These are important omissions and even though regions with CPMHTs may incur higher costs, the benefits in terms of potential increases in the woman’s quality of life, the infant’s quality of life and development in the long-term, and potential reductions in broader societal costs, may offset this increased cost. Results should be interpreted with these caveats in mind.

### Future research recommendations

To be able to fully evaluate the impact of CPMHTs, future research should focus on estimating the short- and long-term impacts on mental health and quality of life outcomes of women who received care in areas with CPMHTs, compared to women in areas without CPMHTs. Furthermore, given that the majority of societal costs associated with perinatal depression, anxiety and psychosis is attributable to the additional long-term healthcare needs of the baby, future research should explore the long-term outcomes for babies born to women who received care from CPMHTs during the perinatal period. By addressing these research recommendations, the full clinical and economic impact of CPMHTs can be assessed.

## Conclusions

Following the implementation of CPMHTs, the percentage of women using acute care decreased while the percentage of women using community care increased. The overall reduction in acute care was driven by a decrease in the number of CRT contacts in areas with CPMHTs, whereas an increase in inpatient admissions was observed in these areas. Overall, the greater use of inpatient admissions alongside greater use of community care resulted in a significantly higher mean cost of secondary mental health service use over the perinatal period for women in the CPMHT group compared with no CPMHT. Increased costs must be considered with caution as we were unable to present this alongside relevant outcomes such as quality of life or satisfaction with services.

### Electronic supplementary material

Below is the link to the electronic supplementary material.

Supplementary Material 1

## Data Availability

This work uses data that has been provided by patients as part of their care and support. The data are collated, maintained and quality assured by NHS Digital, now part of NHS England. Requests for access to these data should be directed to the Data Access Request Service, which is part of NHS England (https://digital.nhs.uk/services/data-access-request-service-dars).
