# Supplementary material for: An exploration of service use pattern changes and cost analysis following implementation of community perinatal mental health teams in pregnant women with a history of specialist mental healthcare in England: a national population-based cohort study
Source: BMC Health Serv Res. 2024 Mar 20;24:359. doi: 10.1186/s12913-024-10553-8 (PMC10983755; doi:10.1186/s12913-024-10553-8)
Supplement: Supplementary file 1 — Supplementary Material 1 [file 12913_2024_10553_MOESM1_ESM.docx]

**Supplementary material**

**Data cleaning assumptions**

**Cluster days assumptions**

- If a cluster is missing an end date and another cluster (with the same start date and care cluster assignment) has an end date we assumed the end date of the cluster without missing data.
- For start date data before the 1st April 2016 in MHSDS we truncate to the 1st April 2016.
- Where clusters with the same cluster assignment partially overlap, we assume these are one care cluster and combine.
- Where a cluster starts the day after another cluster of the same assignment ends, we assume these as one care cluster and combine.
- If a cluster has a missing end date and there is a new cluster (with a different cluster assignment) we assume the end date of the missing cluster is the day before the start date of the new cluster.
- Where two cluster end dates are the same, we assume the start date of the newer cluster is the end date of the older cluster.
- Where an end date of a cluster is after the start date of a newer cluster, we assume the end date of the older cluster is the same as the start date of the newer cluster.
- If a cluster has a missing end date at the end of the reporting period, we use the cluster flag to check if cluster is ongoing. If cluster is ongoing at the end of reporting period we keep as missing, if cluster has ended by the end of the reporting period, we assume an end date of the appropriate reporting period (i.e. 31st March 2017, 2018 or 2019).
- If a cluster date is present and the cluster assignment is missing we have replaced with cluster 99 (patients not assessed or clustered)

**Admitted days assumptions**

- If a spell is missing a discharge date and another spell (with the same admission date) has an admission and discharge date, we assume the discharge date of the spell without missing data.
- For start date data before the 1st April 2016 in MHSDS we truncate to the 1st April 2016.
- If a spell is missing a discharge date but has a planned discharge available, we replace the missing discharge date by planned discharge date.
- Where spells partially or completely overlap, we assume these are one admission and combine.
- Where a spell starts the day after another spell ends, we assume these are one spell and combine.
- If a spell is missing a discharge date (and planned discharge date is not available) within last three months of data end (i.e. 1st January 2019 to 31st March 2019) we assume the admission is ongoing and replace discharge date with end of study period (i.e. 31st March 2019).

**Supplementary table 1: Unit costs**

| **Resource** | **Unit** | **Unit cost 2018/19** | **Source** | **Notes** |
| --- | --- | --- | --- | --- |
| Crisis resolution team | Per contact | £201.87 | Curtis & Burns, 2016 | Inflated from 2016 prices |
| Community mental healthcare | Per contact | £40.36 | Curtis & Burns, 2017 | Inflated from 2017 prices |
| Psychiatric inpatient |  |  |  |  |
| Cluster 00: Variance (unable to assign mental health care cluster code) | Per occupied bed day | £383 | NHS Reference costs 2019 |  |
| Cluster 01: Common mental health problems (low severity) | Per occupied bed day | £371 | NHS Reference costs 2019 |  |
| Cluster 02: Common mental health problems (low severity with greater need) | Per occupied bed day | £408 | NHS Reference costs 2019 |  |
| Cluster 03: Non-psychotic (moderate severity) | Per occupied bed day | £405 | NHS Reference costs 2019 |  |
| Cluster 04: Non-psychotic (severe) | Per occupied bed day | £399 | NHS Reference costs 2019 |  |
| Cluster 05: Non-psychotic (very severe) | Per occupied bed day | £414 | NHS Reference costs 2019 |  |
| Cluster 06: Non-psychotic disorders of over-valued ideas | Per occupied bed day | £411 | NHS Reference costs 2019 |  |
| Cluster 07: Enduring non-psychotic disorders (high disability) | Per occupied bed day | £412 | NHS Reference costs 2019 |  |
| Cluster 08: Non-psychotic chaotic and challenging disorders | Per occupied bed day | £422 | NHS Reference costs 2019 |  |
| Cluster 10: First episode psychosis | Per occupied bed day | £428 | NHS Reference costs 2019 |  |
| Cluster 11: Ongoing recurrent psychosis (low symptoms) | Per occupied bed day | £401 | NHS Reference costs 2019 |  |
| Cluster 12: Ongoing or recurrent psychosis (high disability) | Per occupied bed day | £407 | NHS Reference costs 2019 |  |
| Cluster 13: Ongoing or recurrent psychosis (high symptom and disability) | Per occupied bed day | £414 | NHS Reference costs 2019 |  |
| Cluster 14: Psychotic crisis | Per occupied bed day | £469 | NHS Reference costs 2019 |  |
| Cluster 15: Severe psychotic depression | Per occupied bed day | £406 | NHS Reference costs 2019 |  |
| Cluster 16: Dual diagnosis | Per occupied bed day | £420 | NHS Reference costs 2019 |  |
| Cluster 17: Psychosis and affective disorder (difficult to engage) | Per occupied bed day | £411 | NHS Reference costs 2019 |  |
| Cluster 18: Cognitive impairment (low need) | Per occupied bed day | £492 | NHS Reference costs 2019 |  |
| Cluster 19: Cognitive impairment or dementia (moderate need) | Per occupied bed day | £502 | NHS Reference costs 2019 |  |
| Cluster 20: Cognitive impairment or dementia (high need) | Per occupied bed day | £498 | NHS Reference costs 2019 |  |
| Cluster 21: Cognitive impairment or dementia (high physical or engagement) | Per occupied bed day | £477 | NHS Reference costs 2019 |  |
| Cluster 99: Patients not assessed or clustered | Per occupied bed day | £326 | NHS Reference costs 2019 |  |

**Supplementary table 2: Participant characteristics for those with full economic data versus the full sample**

| Characteristic | Sample with full economic data  (n=70,082) | Sample without full economic data  (n=241) |
| --- | --- | --- |
| Maternal age categories n (%) |  |  |
| 18 to 24 | 17,404 (24.83) | 59 (24.48) |
| 25 to 34 | 39,912 (56.95) | 119 (49.38) |
| 35 to 39 | 10,167 (14.51) | 49 (20.33) |
| 40 and over | 2,593 (3.70) | 14 (5.81) |
| Missing | 6 (0.01) | 0 |
| Obstetric history n (%) |  |  |
| Nulliparous | 22,568 (32.20) | 97 (40.25) |
| Multiparous, no previous CS | 35,315 (50.39) | 108 (44.81) |
| Multiparous, previous CS | 8,627 (12.31) | 19 (7.88) |
| Missing | 3,572 (5.10) | 17 (7.05) |
| Ethnicity n (%) |  |  |
| White | 54,808 (78.21) | 157 (65.15) |
| South Asian | 3,224 (4.60) | 20 (8.30) |
| Black | 1,761 (2.51) | 18 (7.47) |
| Mixed | 1,328 (1.89) | 4 (1.66) |
| Other stated | 1,151 (1.64) | 9 (3.73) |
| Missing | 7,810 (11.14) | 33 (13.69) |
| Socioeconomic deprivation n (%) | |  |
| Quintile 1 (least deprived) | 7,353 (10.49) | 20 (8.30) |
| Quintile 2 | 9,738 (13.90) | 27 (11.20) |
| Quintile 3 | 12,518 (17.86) | 42 (17.43) |
| Quintile 4 | 16,473 (23.51) | 49 (20.33) |
| Quintile 5 (most deprived) | 23,997 (34.24) | 103 (42.74) |
| Missing | 3 (0.00) | 0 |
| Pregnancy risk factors n (%) |  |  |
| Pre-existing diabetes | 1,015 (1.45) | 6 (2.49) |
| Missing | 3,572 (5.10) | 17 (7.05) |
| Pre-existing hypertensive conditions | 487 (0.69) | 1 (0.41) |
| Missing | 3,572 (5.10) | 17 (7.05) |
| Gestational diabetes | 4,273 (6.10) | 21 (8.71) |
| Missing | 3,572 (5.10) | 17 (7.05) |
| Pre-eclampsia | 1,434 (2.05) | 8 (3.32) |
| Missing | 3,572 (5.10) | 17 (7.05) |
| Highest level of pre-pregnancy contact | |  |
| Psychiatric inpatient | 3,272 (4.67) | 121 (50.21) |
| Crisis resolution team | 13,776 (19.66) | 56 (23.24) |
| Community healthcare | 53,034 (75.67) | 64 (26.56) |
| Timing of most recent pre-pregnancy contact | | |
| > 5 years | 18,292 (26.10) | 10 (4.15) |
| 1-5 years | 34,612 (49.39) | 60 (24.90) |
| < 1 years | 17,178 (24.51) | 171 (70.95) |
